# RTX Toxins Ambush Immunity’s First Cellular Responders

**DOI:** 10.3390/toxins11120720

**Published:** 2019-12-10

**Authors:** Laura C. Ristow, Rodney A. Welch

**Affiliations:** Department of Medical Microbiology and Immunology, University of Wisconsin-Madison, Madison, WI 53706, USA

**Keywords:** RTX (repeats-in-toxin) toxin family, β_2_ integrins, Gram-negative bacteria, virulence factor

## Abstract

The repeats-in-toxin (RTX) family represents a unique class of bacterial exoproteins. The first family members described were toxins from Gram-negative bacterial pathogens; however, additional members included exoproteins with diverse functions. Our review focuses on well-characterized RTX family toxins from *Aggregatibacter* *actinomycetemcomitans* (LtxA), *Mannheimia* *haemolytica* (LktA), *Bordetella pertussis* (CyaA), uropathogenic *Escherichia coli* (HlyA), and *Actinobacillus pleuropneumoniae* (ApxIIIA), as well as the studies that have honed in on a single host cell receptor for RTX toxin interactions, the β_2_ integrins. The β_2_ integrin family is composed of heterodimeric members with four unique alpha subunits and a single beta subunit. β_2_ integrins are only found on leukocytes, including neutrophils and monocytes, the first responders to inflammation following bacterial infection. The LtxA, LktA, HlyA, and ApxIIIA toxins target the shared beta subunit, thereby targeting all types of leukocytes. Specific β_2_ integrin family domains are required for the RTX toxin’s cytotoxic activity and are summarized here. Research examining the domains of the RTX toxins required for cytotoxic and hemolytic activity is also summarized. RTX toxins attack and kill phagocytic immune cells expressing a single integrin family, providing an obvious advantage to the pathogen. The critical question that remains, can the specificity of the RTX-β_2_ integrin interaction be therapeutically targeted?

## 1. Introduction

One of the earliest defined exoprotein secretion pathways in bacteria is the mechanism utilized by the repeats-in-toxin (RTX) family. The prototype member of the family is the *Escherichia coli* hemolysin (HlyA) with a primary sequence that contains nonapeptide repeats, a feature that lent to the name of the family [1,2,3]. The family was initially remarkable in that it lacked two aspects characteristic of secreted proteins that used the ubiquitous *sec* machinery in Gram-negative bacteria: an N-terminal cleavable signal sequence and a detectable periplasmic intermediate on its route to the extracellular environment [1,4]. Additional distinguishing features of the family include that the extracellular secretion signal resides in the carboxy-terminal 27–50 amino acids and that there are three transport proteins, HlyB, HlyD, and TolC, necessary for the extracellular secretion process [5,6,7]. Later when it became apparent that the RTX family included a variety of extracellular proteins that had functions other than toxins, such as proteases, lipases, and nodulation factors, Salmond and Reeves proposed a more generic term, Type I Secretory Proteins [8].

The cytolytic activity of RTX toxins found in a wide variety of pathogens requires several notable characteristics. The common host cell damaging event attributed to the RTX toxins is the creation of a membrane pore [9,10,11,12,13]. The toxin proteins are converted from cytotoxically inactive to active forms via post-translational modification by a dedicated acyl transferase (e.g., HlyC) that adds long chain fatty acids to one or two lysine residues [14,15,16]. Lastly, the RTX cytotoxic activity requires that there are Ca^2+^ ions bound to the canonical nonapeptide repeats, of varying quantity in RTX toxin-containing pathogens [17,18,19]. We suggest that the interested reader consult recent reviews that cover in depth the structure, function, secretion, and pathogenic mechanism of the RTX toxins [20,21,22]. In this presentation, we will focus on the topic of host cell receptors that these toxins appear to use to initiate interaction with host cells.

## 2. Host Cell Interactions

### 2.1. Proposed Receptors on Nucleated Cells

**Figure 1:**
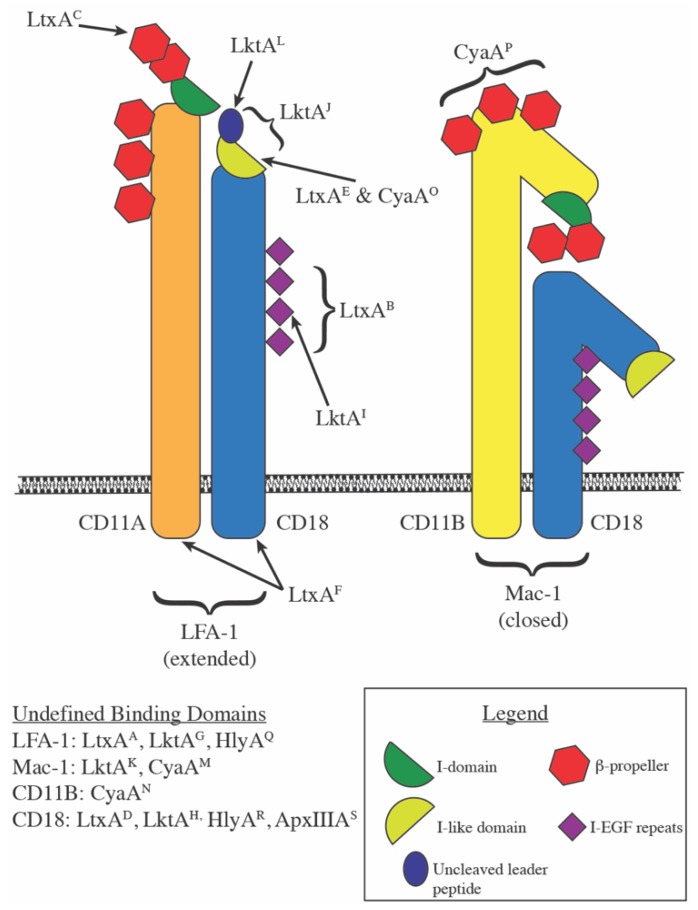
Repeats-in-toxin (RTX) toxin binding sites on β_2_ integrins.

Dozens of studies to identify a receptor for various members of the RTX toxin family have converged on a single receptor family, the β_2_ integrins. Integrins are heterodimeric cell surface receptors involved in a variety of interactions from direct cell-to-cell binding, cell to extracellular matrix interactions, and cell receptor to extracellular soluble ligand interactions. The β_2_ family of integrins is expressed exclusively on leukocytes and encompasses four members, each composed of a unique alpha subunit non-covalently partnered with the β_2_ subunit; CD11A/CD18 (α_L_β_2_, LFA-1 (lymphocyte function-associated antigen-1)), CD11B/CD18 (α_M_β_2_, Mac-1 (macrophage-1 antigen), Complement Receptor 3 (CR3)), CD11C/CD18 (α_X_β_2_, p150/95), and CD11D/CD18 (α_D_β_2_) [23]. Five RTX toxins have been the subject of intense study regarding receptor/toxin interactions and this review will focus on the evidence published over the last 20+ years for the RTX toxins from *Aggregatibacter* (formerly *Actinobacillus*) *actinomycetemcomitans* (LtxA), *Mannheimia* (formerly *Pasteurella*) *haemolytica* (LktA), *Bordetella pertussis* (CyaA), uropathogenic *Escherichia coli* (HlyA), and *Actinobacillus pleuropneumoniae* (ApxIIIA) (Figure 1).

Structural models of LFA-1 and Mac-1, respectively composed of CD11A/CD18 and CD11B/CD18, are shown with labels A through S indicate where potential binding sites for RTX toxins occur, with the published reference for the indicated binding site detailed below.

(A) LtxA: LFA-1 [24]
(B) LtxA: CD18 EGF-like domains 2, 3, and 4 within amino acids 500–600 [25]
(C) LtxA: CD11A β-sheets 1 and 2 of the β-propeller region [26,27]
(D) LtxA: CD18 [28,29]
(E) LtxA: Activated LFA-1 (m24 epitope) [30]
(F) LtxA: CD11A and CD18 cytoplasmic tails [31]
(G) LktA: LFA-1 [32,33,34,35,36]
(H) LktA: CD18 [32,33,37,38,39,40]
(I) LktA: CD18 EGF-like domain 3 within amino acids 500–600 [41,42]
(J) LktA: CD18 amino acids 1–291 [43]
(K) LktA: Mac-1 [44]
(L) LktA: CD18 uncleaved signal peptide [45]
(M) CyaA: Mac-1 [46]
(N) CyaA: CD11B
(O) CyaA: Activated LFA-1 (m24 epitope) [47]
(P) CyaA: CD11B amino acids 614-682 [48]
(Q) HlyA: LFA-1 [24]
(R) HlyA: extra-cytoplasmic region of CD18 [29]
(S) ApxIIIA: CD18 [49]

#### 2.1.1. LtxA

In 1997, Ned Lally and his colleagues published the first description of CD11A/CD18 as a receptor for LtxA [24]. The group took an unbiased approach to receptor identification when they generated and isolated monoclonal antibodies against the susceptible human leukemic cell line, HL-60, and assayed the antibodies for the ability to inhibit cytolytic activity of LtxA. One of the neutralizing antibodies, mAb295, was used to immunopurify targets from HL-60 cell lysates. Tryptic peptides were generated from two interacting species, 170 and 100 kDa, respectively, which led to the identification of CD11A and CD18. With specific targets characterized, additional monoclonal antibodies against CD11A and CD18 were interrogated for inhibition of toxin cytolytic activity. Results varied across different antibodies described in the publication, with the highest inhibition resulting from preincubation of cells with a monoclonal anti-CD18 antibody (KIM185), inhibiting 90% of the LtxA cytotoxic activity. To functionally confirm the interaction of LtxA with CD11A/CD18, a gain of sensitivity was demonstrated by ectopically expressing CD11A and CD18 in K562 cells, a human erythroleukemic cell line, which enhanced the cytolytic activity of LtxA compared to parental K562 cells.

To dissect LFA-1 to identify the specific subunit and interacting domain for LtxA, the human specificity of the toxin was exploited by generating human/bovine CD11A/CD18 heterodimers and ectopically expressing these constructs in K562 cells [25]. Only cell lines that expressed human CD18 were sensitive to LtxA activity. This observation led to the construction of human/bovine CD18 chimeras to investigate the specific domains of CD18 required for LtxA activity. With a panel of 13 chimeras, the authors described that the EGF (epidermal growth factor)-like domains 2, 3, and 4 within amino acids 500–600 of CD18 provide the species specificity for LtxA.

Only a few months later, a thorough examination of the species specificity of the interaction of LtxA with β_2_ integrins was published by Kieba et al. [26]. The human T-cell line, Jurkat, is sensitive to LtxA, whereas a mutant derivative that lacks CD11A expression (J-β_2_.7) is resistant to the cytolytic activity of the toxin [26,50]. Complementation of J-β_2_.7 cells with human, bovine, or mouse CD11A constructs restored surface expression of the heterodimeric LFA-1, but only human and bovine complementation restored sensitivity to LtxA. The lack of sensitivity in the murine CD11A complement was exploited to determine which portions of human CD11A conferred LtxA sensitivity by constructing chimeric human/murine CD11A constructs. Complements that remained sensitive contained at minimum the first 128 amino acids of human CD11A and led to the conclusion that beta sheets 1 and 2 of the propeller region of human CD11A are required for LtxA sensitivity.

In 2013, Reinholdt et al. published the first report that investigated additional β_2_ integrin family members competent for LtxA-mediated cytotoxic effects, although their research was focused on optimizing a novel purification method for LtxA [28]. The authors isolated LPS-free LtxA in its native conformation and examined the cytolytic and hemolytic activity of the toxin. In contrast to previous studies, the authors expanded the repertoire of β_2_ integrin species examined and generated cell lines ectopically expressing human CD11A/CD18, CD11B/CD18, and CD11C/CD18. All three cell lines were equally susceptible to purified LtxA. Direct binding of LtxA to the integrin subunits was investigated in vitro with far-western blots, a method in which cell membrane extracts from the cell lines described above (ectopically expressing integrin subunits) were separated by SDS-PAGE and transferred to a membrane. The membrane was incubated with soluble LtxA and bound LtxA was detected as in a traditional western blot. LtxA bound only to the band that corresponded to the β_2_ portion of the integrin, none of the alpha subunits. To this point, although the early papers reviewed here report that CD11A/CD18 is the only receptor for LtxA, it could be explained by the cell lines in use. HL-60 cells were used by Lally et al. to first characterize binding partners of LtxA [24]. Undifferentiated HL-60 cells only express LFA-1 of the β_2_ integrin family and so both the exclusive pulldown of CD11A/CD18 with LtxA and the ability of certain CD11A/CD18 antibodies to neutralize LtxA activity are not surprising [23]. Follow-up studies ectopically expressed only CD11A/CD18 in non-susceptible cell lines and Lally et al. confirmed the lack of endogenous CD11B and CD11C surface expression on K562 cells, so the conclusions could only be related to the interaction of LtxA with LFA-1. In light of the Reinholdt et al. results that LtxA is equally cytotoxic to all β_2_ integrin species tested and that direct binding only occurs with the β_2_ subunit, the previous identification of the β-propeller region of CD11A as required for LtxA activity and recent results from Krueger et al. are seemingly contradictory [26,27].

Based on the Kieba et al. publication, Krueger et al. generated peptides which mimic the proposed binding region of LtxA on the CD11A subunit to inhibit LtxA activity [27]. Preincubation of LtxA with the peptides inhibited cytolytic activity on THP-1 cells (human monocytes). Undifferentiated THP-1 cells are similar to HL-60 cells in that CD11A/CD18 is the most highly expressed β_2_ integrin, with low levels of CD11C/CD18 also present, so it is likely that complete blocking of only CD11A/CD18 blocks a significant portion of the total population of β_2_ family integrins and could significantly reduce the cytolytic effects of LtxA [51]. Although the direct interaction of LtxA with CD18 was the only observed band by far-western, it is possible that the physical interaction of LtxA with CD11A is not optimal on the static membrane and so the conflicting results at first glance may be a result of the experimental limitations in each publication. 

DiFranco et al. report that LtxA preferentially interacts with activated LFA-1 [30]. Primary human white blood cells or THP-1 cells were activated with TPA (12-O-tetradecanoylphorbol-13-acetate), which exposes a monoclonal antibody epitope (m24). Treatment of activated primary cells with LtxA resulted in a shift of m24+ cells to m24− cells. The authors confirm that this is due to LtxA interference with the antibody epitope and not LtxA returning the integrin to the resting state by activating and fixing THP-1 cells, then assessing accessibility of the m24 epitope before and after toxin treatment. LtxA effectively blocks the m24 epitope on activated THP-1 cells.

Our lab recently reported that CD18 is sufficient for LtxA activity [29]. This study was performed in undifferentiated U937 cells, a human monocytic cell line, which express at least three of the four β_2_ integrin heterodimers [51]. Following the identification of the CD18 encoding gene (*ITGB2*) as involved in the cytolytic activity of UPEC HlyA from a library of CRISPR/Cas9 mutants in the U937 background, individual β_2_ integrin family subunit knockout cell lines were constructed, as well as a cell line with all four alpha subunits knocked out. As a related RTX toxin, we examined the role of β_2_ integrins in LtxA cytolytic activity and found that similar to HlyA, all four of the alpha subunits were redundant for LtxA activity, whereas in cell lines devoid of CD18, LtxA was completely inactive. We reproduced the far-western result published in Reinholdt et al., in that LtxA bound to CD18, but not CD11A, CD11B, or CD11C on a nitrocellulose membrane. We also complemented CD18 deficient U937 cells with wild-type CD18 or a cytoplasmic tail deficient CD18 and found that both restored wild-type sensitivity to LtxA, indicating that intracellular signaling via the cytoplasmic tail is not required for the cytolytic activity. Nygren et al. recently published that LtxA activates CD11A/CD18 on the surface of the cell and upon internalization, interacts with the cytoplasmic tails of CD11A and CD18, but not CD11B or CD11C [31]. The affinity of LtxA for the cytoplasmic tails was measured by surface plasmon resonance (SPR) and although we demonstrated that the cytoplasmic tail of CD18 is not necessary for the cytolytic activity of LtxA, interaction may still occur with unidentified downstream effects.

With the exclusive expression of β_2_ integrins on leukocytes, the potential for therapeutic use of LtxA was quickly recognized. As early as 2010, publications demonstrating the efficacy of LtxA as a therapeutic treatment have been reported and LtxA has been trademarked as “Leukothera®” the applications and clinical trials for which have been reviewed recently [52,53]. A report focused on legitimizing the rodent model for investigations of LtxA therapeutic use provided an in vivo look at the effects of LtxA cytolytic activity on leukocytes. In vivo injection of LtxA reduces the total white blood cell count by 12 min post-injection in CD-1 mice [54]. The specificity of LtxA for CD11A/CD18 was concluded after injection of LtxA in C57Bl/6 parental mice reduced total white blood cells 2–3-fold compared to untreated mice, whereas total white blood cell counts were unchanged in LtxA-treated or untreated C57Bl/6 CD11A knockout mice. To further establish the model system and expand the techniques that could be utilized, the group examined the sensitivity of murine cell lines to LtxA for in vitro assays, in a myeloblastic leukemia (M1) and mouse lung epithelial cell (MLE). Epithelial cells are predicted to have no β_2_ integrin expression, so the result that the MLE cells were more resistant to LtxA activity than M1 cells was expected; however, as a minor point, they did observe 15% cell death at the highest LtxA concentrations on MLE cells (10% cell death observed in untreated cells at the same time point). Even the more sensitive mouse line was considerably less sensitive than human THP-1 cells and was never 100% sensitive to LtxA at the highest concentrations of toxins tested. The in vitro death of ~60% of murine cells aligns with the 2–3-fold reductions of white blood cells observed in vivo. The significant differences in LtxA activity on murine cells compared to human cells remain an issue for translating results in the rodent model system to human therapeutic use.

#### 2.1.2. LktA

Reports describing the interaction of LktA with the β_2_ integrin family are conflicted as well. One of the earliest publications regarding the mode of action for LktA delved into the effects of low compared to high concentration LktA treatment on BL-3 cells, a bovine lymphocyte cell line. Low concentrations of LktA induced apoptosis and could be neutralized with one of two anti-LFA-1 antibodies interrogated and an anti-CD18 antibody, providing the first evidence for this RTX toxin’s interaction with the β_2_ integrin family [33]. Several monoclonal anti-CD11B and CD11C antibodies were tested with no inhibition of LktA activity observed. Solubilized BL-3 cells were passed over immobilized LktA on polystyrene beads and bound proteins were characterized. The bovine anti-LFA-1 monoclonal antibody bound to a single molecular weight band in whole BL-3 lysates and LktA-bound proteins from BL-3 lysates, corresponding to the molecular weight of CD18 alone. Although this result indicates that LktA binds CD18, it does not characterize the alpha subunits on the same blots to assess whether they were also LktA-bound following incubation of LktA with BL-3 lysates.

Within a year, two publications described the interaction of LktA with CD18 alone. The first report used ligand blotting of BL-3 cell membranes separated by SDS-PAGE with LktA to identify interacting partners [39]. The two bands that bound LktA were 95 and 100 kDa and parallel blots revealed that bovine anti-CD18 or anti-CD11A/CD18 antibodies identified the two bands. The anti-CD18 antibody was capable of inhibiting ~55% of LktA cytolytic activity on BL-3 cells. Ambagala et al. immobilized LktA to identify interacting partners from a host cell lysate, similar to the initial report describing LktA interaction with β_2_ integrins, but with a bovine polymorphonuclear leukocyte (PMN)-lysate [37]. Four polypeptides were isolated and characterized as CD11A, CD11B, CD11C, and CD18. A bovine specific anti-CD18 antibody reduced the cytolytic activity of LktA on PMNs 2-fold, while monoclonal antibodies against CD11A, CD11B, CD11C, or LFA-1 were not neutralizing. In this report, the alpha subunits for CD18 were pulled down with LktA and characterized, but dismissed as specific receptors as the alpha subunit specific antibodies were not neutralizing against LktA activity.

The back and forth continued in 2000 with a report that only CD11A with CD18 enhances LktA activity [35]. In this publication, cell lysates from bovine PMNs, bovine macrophages, porcine alveolar macrophages, and human monocytic cells were assessed for binding to LktA-coated beads. LFA-1 was the only β_2_ integrin pulled down, despite the expression of CD11B and CD11C in the bovine and porcine cells, confirmed by flow cytometry. Although LktA bound to LFA-1 from porcine cells, the cells were resistant to LktA-mediated cytolytic activity. LktA did not bind to β_2_ integrins in the human cell line, which are resistant to LktA activity. Cell pretreatment with anti-CD11A or anti-CD18 could neutralize LktA binding and subsequent LDH release in both types of bovine cells examined. The authors also tested two unique anti-CD11B antibodies and an anti-CD11C antibody, but observed no neutralization. In support of these findings, pretreatment with bovine IL-1β, a novel technique for upregulating β_2_ integrins on bovine neutrophils, enhanced LktA activity [36]. Pretreatment with an anti-LFA-1 antibody on β_2_ integrin-upregulated neutrophils neutralized LktA cytolytic activity.

In 2002, Deshpande et al. demonstrated both necessity and sufficiency of CD18 for LktA mediated cytolytic activity by expression of bovine CD18 in a LktA-resistant murine mastocytoma cell line, P815 [38]. A stable transfectant (2B2) paired bovine CD18 with murine CD11A on the surface and was sensitive to LktA in a concentration-dependent manner, with 75% of cells killed at the maximum LktA concentration used. This gain of sensitivity could be inhibited partially (30%) with an anti-bovine CD18 monoclonal antibody.

Three publications came from the same group in 2005 with a progression of evidence for CD18 serving as the sole receptor for LktA: Thumbikat et al. (Vet Res), Dileepan et al. (Microbiol Pathogen), and Dileepan et al. (Infect Immun) [34,40,41]. In the first publication, the authors demonstrate that LktA can bind to both Mac-1 or LFA-1 on bovine macrophages, but that intracellular signaling, i.e., phosphorylation of the CD18 tail, only occurs downstream of the interaction of LktA with LFA-1 [40]. In the second paper, a LktA resistant human erythroleukemic cell line, K562 cells, was used to ectopically express bovine LFA-1 and demonstrate direct interaction of LktA with LFA-1 [34]. Cell lysates were generated from the transduced cells, depleted of either CD11A or CD18 and the remaining proteins were incubated with LktA-coated beads to determine interacting partners. In both types of depletion, LktA was able to interact with the remaining partner of the β_2_ integrin. In the third publication, a series of bovine/human CD18 chimeric recombinant proteins were ectopically expressed in K562 cells and the only cell lines that were susceptible to the LktA toxin were those that contained the EGF-like domains present within the CD18 500–600 amino acid region [41].

Within a month of the final publication of the trio above, a report defined a unique region of bovine CD18 for interaction with LktA. This publication also used interspecies hybrid CD18 ectopic expression, but built from the murine cell lines that ectopically express LFA-1 (P815 cells) reported previously [38,43]. Upon expression of bovine CD18, these cells are sensitized to LktA. With chimeric murine/bovine forms of CD18, they narrowed the region of CD18 required for LktA cytolytic activity to the N-terminus of the protein, amino acids 1–291. Two years later, Dileepan et al. had built on the evidence in their 2005 reports by generating additional chimeras between human and bovine CD18 to narrow the domain required for sensitivity to LktA to bovine CD18 residues 541–581, which contains EGF-like-domain 3 [42]. In the midst of the flurry of papers describing the regions of CD18 required for sensitivity to LktA, a publication ectopically expressed bovine, sheep or Bighorn sheep forms of CD11B/CD18 on human epithelial kidney cells (HEK293) and sensitized those cells to LktA, reinforcing the ruminant specificity of LktA as well as providing evidence that CD11B/CD18 is competent as a receptor, not only CD11A/CD18 [44].

In 2007, a publication entitled “Monomeric Expression of Bovine β_2_-Integrin Subunits Reveals Their Role in *Mannheimia haemolytica* Leukotoxin-Induced Biological Effects”, went to considerable effort to generate cell lines that ectopically express individual integrin subunits on the surface [32]. Canonically, integrins are described to act as heterodimers and require a binding partner for proper processing and expression on the surface of cells, although a handful of groups have reported expression of single integrin subunits on transfected cells in either the CHO (Chinese hamster ovary) or HEK293 human cell line backgrounds [48,55,56,57]. The authors of this publication report that they required sequential enrichment by magnetic bead-antibody sorting of transductants to obtain individual integrin subunit expression on the surface. Once these clones were obtained, with CD11A alone, CD18 alone, or CD11A/CD18 heterodimers, they demonstrated that LktA bound to all three cell types. Although binding occurs, only ~15% of CD11A-expressing cells are killed when treated with LktA, similar to the parental HEK293 cells, whereas CD18 alone or CD11A/CD18 heterodimer-expressing cells are ~50% sensitive to the highest concentrations of LktA tested.

In 2009, synthetic peptides based on the bovine CD18 signal peptide inhibited LktA cytolytic activity in BL-3 cells [45]. The authors found that a cleavage resistant glutamine residue is conserved across ruminants at the -5 position in the unprocessed bovine CD18. Substitution of this residue to a glycine resulted in cleavage of CD18 and LktA treated cells were resistant to the cytolytic activity of the toxin. These observations were translated to bovine clinical research in a subsequent publication in which a bovine fetus was genetically engineered to express the mutated CD18 allele. Leukocytes isolated from this fetus are resistant to LktA cytolytic activity and provide promise to the potential to genetically engineer cattle that are resistant to *Mannheimia haemolytica* pneumonia [58]. This in vivo demonstration of the importance of CD18 alone provides strong support for the publications described above which conclude that CD18 alone is the critical binding partner for LktA cytolytic activity.

#### 2.1.3. CyaA

The bi-functional adenylate cyclase/RTX toxin from *Bordetella pertussis* was the first RTX toxin reported to interact with CD11B/CD18, in 2001 [46]. Following the reports of other leukocyte-specific RTX toxins utilizing the β_2_ integrin family, four mouse cell lines were screened for CyaA binding, with the hypothesis that the cell line with the most saturable binding would express the most receptors. Indeed, they demonstrated that D1 cells, a splenic myeloid dendritic cell line, expressed the most CD18 and bound the greatest amount of CyaA. On the cell line with the second highest binding, J774, a mouse macrophage cell line, they demonstrated that anti-CD11B monoclonal antibodies inhibited CyaA-binding and consequent LDH release, but monoclonal antibodies against CD11A, CD11C, or CD18 did not. Ectopic expression of CD11B/CD18 or CD11C/CD18 in CHO cells was demonstrated to sensitize only those cells expressing CD11B. A subsequent publication from the same group utilized the CD11B/CD18 expressing CHO cells to demonstrate the ability of anti-CD11B antibodies to neutralize CyaA before toxin addition or during simultaneous antibody/toxin treatment [59]. The glycosylation state of CD11B/CD18 is important for CyaA activity, as treatment of either natively CD11B/CD18-expressing cell lines or CHO cells ectopically expressing the receptor with glycosidases or flooding the experimental environment with free sugars increased resistance to CyaA activity [60]. To further investigate the required portions of CD11B for interaction with CyaA and cement the importance of this alpha subunit compared to other family members, Osicka et al. constructed chimeras of CD11B and CD11C and localized the binding site to amino acids 614–682 of CD11B [48]. This region of CD11B contains the β-propeller domain C-terminal to the I-domain. Binding of CyaA to CD11B does not activate Syk phosphorylation, as do native ligands of the I-domain, supporting the unique binding location of the toxin. CyaA does not bind CHO cells expressing CD11A or CD11C, providing additional evidence that CD11B is the only receptor for CyaA on leukocytes.

In the midst of these publications, a second group published that CyaA can bind to CD11A on primary peripheral blood T lymphocytes [47]. CyaA bound resting T lymphocytes in a saturable manner. If the cells were activated with an anti-CD3 monoclonal antibody, CyaA binding increased four-fold compared to inactive lymphocytes. To investigate the interaction of CyaA with CD11A, cells were treated with an anti-CD11A monoclonal antibody (HI111) on ice, then shifted to 37 °C, which induces internalization of LFA-1. This treatment regimen results in an 80% reduction in CyaA binding to the cells. Additionally, if LFA-1 was induced to a high affinity state, an epitope becomes accessible to a unique monoclonal anti-CD11A antibody (m24) and pretreatment with this antibody can block CyaA binding. In CD11A-deficient Jurkat cells (J-β_2_.7), originally described from the Klickstein lab, CyaA binds 4–5-fold less than parental Jurkat cells, which is reversed when J-β_2_.7 cells are complemented with CD11A [61]. In contrast to LtxA and LktA, the CD18 subunit has not been characterized as the critical portion of the β_2_ integrins for the binding of CyaA.

#### 2.1.4. HlyA

Unlike the controversies for the RTX toxins discussed above, with back and forth evidence regarding which integrin subunits are required for binding or cytolytic activity, for HlyA the controversy is rooted in the more basic biological question of whether a receptor exists or not. The pioneering publication that described the interaction of LtxA with β_2_ integrins also investigated HlyA [24]. Monoclonal antibodies against CD11A and CD18 inhibited HlyA cytolytic activity in HL-60 cells, with the highest inhibition demonstrated by an anti-CD18 antibody. Ectopic expression of LFA-1 in K562 cells significantly increased sensitivity to HlyA cytolytic activity compared to the parental cell line [24].

Valeva et al. retorted the original description of LFA-1 as a receptor for HlyA by demonstrating that the ectopic expression of LFA-1 on K562 cells enhances sensitivity to not only HlyA, but a number of pore-forming toxins, including streptolysin-O, *Staphylococcus aureus* α-toxin, and *Vibrio cholerae* cytolysin [62]. In addition, competitive binding experiments with HlyA or pro-HlyA (mutants at either acylation site or both) were performed on human polymorphonuclear leukocytes, K562 cells, or K562 cells expressing LFA-1. The collective sum of these experiments demonstrated no advantage for wild-type HlyA over any of the mutant forms of the toxin, or on cells with or without LFA-1. 

A third group observed no increase in sensitivity to HlyA in K562 cells transfected to express LFA-1 [63]. In a publication focused on CyaA, described above, the authors also demonstrate that treatment of Jurkat cells with a cocktail of glycosidases reduces the cytolytic activity of HlyA [60]. The conclusions of this report were based on the premise that HlyA binds LFA-1, and that it is the activity of the glycosidases on LFA-1 that reduces HlyA recognition of/activity on the cells, although the glycosidase activity is non-specific and could target any sugar modifications of surface proteins (potential receptors) on the cells. 

As described for LtxA, we recently demonstrated that CD18 is necessary and sufficient for the cytolytic activity of HlyA [29]. We performed an unbiased genome-wide CRISPR/Cas9 library screen in U937 cells with HlyA for host factors involved in the cytolytic activity of the toxin. Following identification of CD18 (*ITGB2*), cell lines were generated to characterize the involvement of the entire β_2_ integrin family, with individual alpha subunits or the β_2_ subunit knocked out. Knockout of any single alpha subunit or all four alpha subunits simultaneously had no effect on the sensitivity of U937 cells to HlyA. The quadruple alpha subunit mutant cell line revealed that the alpha subunits are not redundant for HlyA activity; they are not required at all. A significant increase in resistance to HlyA was only observed when CD18 was knocked out, as CD18-deficient cells were ~100-fold more resistant to the cytolytic activity of the toxin. In a far-western blot, HlyA bound to CD18, but none of the alpha subunits. CD18-deficient cells complemented with wild-type CD18 or a cytoplasmic tail deficient form of CD18 regained wild-type levels of sensitivity to HlyA, from which we concluded that signaling downstream of the integrin is not required for the cytolytic activity of HlyA. Similar to CyaA, the body of evidence behind the existence of an HlyA receptor is much smaller, but for HlyA appears to favor binding CD18 alone, like LtxA and LktA.

#### 2.1.5. ApxIIIA

A lone report exists describing the involvement of β_2_ integrins with one of the RTX toxins from *A. pleuropneumoniae*, guided by the research described above for the other RTX family members. Vanden Bergh et al. utilized K562 cells to express CD11A/CD18 heterodimers composed of a combination of human, bovine, or porcine subunits [64]. Only those cell lines expressing porcine CD18 were susceptible to ApxIIIA, providing gain-of-function evidence that the leukocyte specificity of ApxIIIA, defined a year earlier by this group, is due to the β-subunit of β_2_ integrins [49]. This report adds ApxIIIA to the expanding list of RTX toxins that utilize CD18 to exhibit cytolytic activity.

### 2.2. Proposed Receptors for Red Blood Cells

The prototypical RTX toxin, *E. coli* hemolysin (HlyA), was so-named because it was initially recognized for the ability to lyse erythrocytes [65]. This lytic activity is not ubiquitous across the RTX family members, as there are canonically two groups of toxins: hemolysins with broad species activity against erythrocytes and nucleated cells, and leukotoxins, with leukocyte specific activity and often narrow species specificity. Although the pore-forming function of the hemolysins is thought to be responsible for the erythrolytic activity, several reports describe additional factors involved in the erythrolytic activity of the hemolysins.

Ostolaza and colleagues demonstrated that glycophorin acts as a receptor on human red blood cells (RBCs) for HlyA [66]. Anti-glycophorin antibodies neutralized HlyA erythrolysis and preincubation of HlyA with purified glycophorin blocked the lytic activity of the toxin. Glycophorin directly bound to immobilized HlyA in a detergent lysate of red blood cells. Early reports of LtxA characterized the toxin as non-hemolytic; however, a comprehensive study of a variety of *Aggregatibacter actinomycetemcomitans* strains and growth media revealed that the hemolytic phenotype is dependent on the source and type of growth medium [67]. LtxA can lyse human and sheep RBCs more efficiently than mouse RBCs, dependent on sialic acid residues and the negative charge imparted by them on the cell surface, as neuraminidase treatment can inhibit LtxA dose-dependent hemolytic activity [63]. Glycophorin is not a receptor for LtxA; however, LtxA appears to bind a variety of glycosphingolipids with sialic acid modifications [68]. LtxA hemolytic activity can be blocked by pretreating the toxin with any of 5 gangliosides: GM1, GM3, GD1a, GD1b, and GT1b.

Purinergic receptors, which mediate signaling by purine nucleotides, indirectly affect the hemolytic activity of HlyA, as inhibitors of P2X_1_ and P2X_7_ do not block pore formation by HlyA, but can inhibit lysis in human, murine, and equine red blood cells [69]. This is also true for LtxA, in that P2X_1_ is required for LtxA induced hemolysis; however, P2X_7_ can enhance, but is not required, for LtxA hemolytic activity on human red blood cells [70].

### 2.3. Receptorless Interactions

Several reports describe unique interactions of RTX toxins with nucleated host cells, independent of β_2_ integrins. Nice et al. recently described the association of LtxA with outer membrane vesicles derived from the *A. actinomycetemcomitans* outer membrane [71]. Up to a third of the total secreted LtxA is associated with outer membrane vesicles, which can associate with host cells independently of characterized receptors/interacting partners like LFA-1 or cholesterol.

Following identification of the role for P2X receptors in RTX toxin activity on erythrocytes, their function in nucleated cells was examined. In 2016, Fagerberg et al. described that inhibitors of P2X_1_, P2X_4_, or P2X_7_ on THP-1 cells or primary human monocytes significantly reduced cell lysis by LtxA or HlyA [72]. For LtxA, inhibitors used on THP-1 cells reduced lysis by ~50%, whereas those same inhibitors on primary monocytes were far less effective. For HlyA, although the inhibition was significant, the authors acknowledge that HlyA was not very active at the concentration (erythrocyte EC_50_) they used on THP-1 cells, so the reduction from 10% death in HlyA treatment alone to 8, 5, or 3% total death in HlyA treatment with various inhibitors could be more convincing.

CyaA can bind to and elevate cAMP levels in epithelial cells, which do not express β_2_ integrins [73]. Specifically, CyaA added to the basolateral membrane of a polarized epithelium, resulting in intoxication, whereas CyaA added to the apical membrane and is completely resistant to the toxin’s effects. 

HlyA is cytolytic at high concentrations to epithelial cells or mutagenized U937 cells that do not express β_2_ integrins [29]. This is in contrast to LtxA, which also requires CD18 for activity, but is completely inactive in the absence of the integrin. Pore formation by RTX toxins has been the subject of critical study for several decades (a thorough review was recently published [74]). It is possible that some of the RTX toxins, like HlyA, possess the ability to form pores in the absence of a receptor that leads to cell death, or that there remain unidentified/secondary receptors, perhaps a lower affinity than the interaction of the RTX toxins with β_2_ integrins that facilitate the cytolytic activity. 

## 3. Toxin Domains Responsible for Receptor Interactions

**Figure 2:**
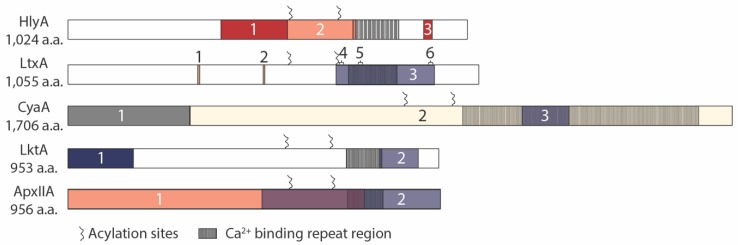
RTX toxin receptor binding sites. HlyA: (1) a.a. 392–563: involved in hemolytic activity [82], (2) a.a. 564–739: required for hemolytic activity [28], (3) a.a. 914–936: glycophorin binding domain [83], acylation sites: K564, K690 LtxA: (1) CRAC domains: 333–339 (CRAC336), (2) 501–505 (CRAC503) [84], (3) a.a. 688–941: required for cytolytic activity on HL-60 cells [85], Neutralizing antibodies: (4) a.a. 698–709, (5) 746–757, (6) 926–937 [36], acylation sites: K562, K687 CyaA: (1) Adenylate cyclase: a.a. 1–312, (2) RTX domain: a.a. 313–1706, (3) a.a. 1166–1287: required for interaction with integrin [59], acylation sites: K860, K983 LktA: (1) a.a. 1–169: involved in species specificity [82], (2) a.a. 800–900: involved in species specificity on BL-3 cells (85), acylation sites: K554, K669 ApxIIA: (1) a.a. 1–762: required for hemolytic activity [86], (2) a.a. 498–956: required for leukocytic activity [56], putative acylation sites: K564, K674.

In contrast to the numerous reports regarding receptors for RTX toxins, there are relatively few publications focused on the RTX toxin domains that interact with putative receptors or the features that are required for cytolytic activity. Several groups have examined the post-translational modification of one or two lysine residues by the “C” gene for each RTX toxin, and determined that this modification is required for full lytic activity (LtxA [75], LktA [76], CyaA [77,78], HlyA [14,15,79]), with two notable exceptions reported. Pro-LktA is cytotoxic to PMNs from Bighorn Sheep, at 128-fold lower levels than mature LktA [80]. A few reports exist of pro-CyaA binding to cells and inducing the same intracellular effects as seen for mature CyaA, i.e., transient cAMP increase, ATP decrease, and annexin V labeling, albeit at 100–1000-fold higher concentrations than the mature form of the toxin [59,73,81]. In addition to acylation, specific domains have been characterized for interaction with host receptors for multiple RTX toxins, with several independent groups capitalizing on the species specificity of LktA for bovine cells to generate gain-of-function chimeric proteins with an additional RTX toxin of interest (Figure 2). 

### 3.1. HlyA

The first report to utilize LktA for chimeric protein construction combined the bovine specific RTX leukotoxin with the ubiquitously cytolytic HlyA. These LktA/HlyA hybrid proteins were created to characterize the regions of the HlyA required for hemolytic and cytotoxic activity [82]. From this study, our lab described that the region between amino acids 564–739 is involved in the lysis of erythrocytes and the region upstream, between amino acids 392–563, has an additional factor that facilitates HlyA hemolytic activity. Ostolaza and co-workers showed that the C-terminal portion of the HlyA toxin, amino acids 914–936, contain the glycophorin binding region [83].

### 3.2. LtxA

Lally et al. also generated chimeric LtxA/LktA proteins to define the species specificity of LtxA. The cytolytic activity of chimeric toxins was assessed on HL-60 cells by trypan blue exclusion [85]. The minimal region of LtxA required for human specific cytolytic activity is conferred by a 253 amino acid sequence between 688 and 941, encompassing the calcium-binding repeats. In addition to chimeric constructs defining this portion of the protein, three LtxA neutralizing antibodies were mapped within this region, spanning amino acids 698–709, 746–757 and 926–937. 

Two cholesterol recognition/amino acid consensus (CRAC) sites were identified in LtxA, CRAC 336 and CRAC 503 [84]. The authors demonstrate that LtxA has a strong and specific affinity for cholesterol, independent of the acylation status of the toxin. Peptides that inhibit the LtxA/cholesterol interaction can inhibit the cytolytic activity of the toxin on Jurkat cells and CRAC336 is sufficient for enhancing the cytolytic activity of the toxin. These domains are shared across several RTX toxins, including LktA, *Actinobacillus pleuropneumoniae* AppA (ApxIIA), CyaA, and HlyA. The authors report that it is shared in *E. coli* HlyA, but upon examination, the CRAC domain they described is found in enterohemorrhagic *E. coli* EhxA and the CRAC domain sequences are not present in the UPEC HlyA. Experimental evidence for the function of these CRAC domains in the additional RTX toxins and whether they are required for cytolytic activity has not yet been reported.

### 3.3. CyaA

The bi-functional nature of CyaA required examination to determine which portion of the toxin provided the interaction with CD11B described above. Binding experiments were performed with the two functional domains of CyaA; the adenylate cyclase and the RTX toxin. The N-terminal CyaA active site fragment did not bind to or block CD11B binding, whereas the hemolytic domain of CyaA 373–1706 bound CD11B. FLAG tag insertions at 17 sites within CyaA revealed that three insertions at 1166, 1245–1273, or 1281 resulted in a loss of the ability to compete for binding with wild-type CyaA or the hemolytic domain of the protein on CD11B-expressing CHO cells. The authors conclude that within the region of 1166–1287 lie critical residues for the interaction of CyaA with the integrin [59].

### 3.4. LktA

Although LktA was used in several studies to characterize the human specificity for other RTX toxins, the regions of LktA required for bovine specificity or cytolytic activity have not been directly examined. Despite not being the focus of these studies, from the HlyA/LktA chimeras, the evidence demonstrates that the N-terminal 169 amino acids of LktA is involved in species specificity, as hybrid proteins containing this region from LktA and the remaining C-terminal region from HlyA were more efficient at killing BL-3 cells than Raji cells [82]. In the LtxA/LktA hybrid proteins, the data suggest the specificity for BL-3 cells lies within the LktA amino acids 800–900 [85]. Additional testing is required to reconcile these seemingly disparate results.

### 3.5. ApxIIA

*A. pleuropneumoniae* encodes four RTX toxins, one of which is described to interact with β_2_ integrins (ApxIIIA [49]). One of the other three toxins, ApxIIA, was examined in 1992 to characterize the domains responsible for the observed hemolytic and cytotoxic activity of the protein. The hemolytic activity of AppA (now designated ApxIIA [87]) is conferred within the domain including the start site to amino acid 762, whereas the leukocytic activity is conferred from within amino acids 498–956 (stop codon) [86].

## 4. Discussion and Conclusions

For the bacterial pathogens that encode RTX toxins, neutrophils and monocytes are the first responders to inflammation induced by infection and their phagocytic activity can play an important role in fighting the bacterial invaders. The targeting of β_2_ integrins by the secreted RTX toxins of these pathogens may have evolved to remotely attack the host immune system and allow the pathogen valuable time to cause disseminated infection. Targeting the universal β_2_ subunit, as four of the five toxins reviewed here do, allows for a single bacterial virulence factor to attack multiple classes of host immune cells. The interacting domain for HlyA on CD18 remains an open question. If it is a unique region on CD18, not overlapping with the EGF domains identified to interact with LtxA and LktA, the identification of convergent evolution within the RTX family would be interesting. In work not reviewed here, studies to characterize the downstream effects of interaction of the RTX toxins with β_2_ integrins are underway in several laboratories and will be critical to our understanding of the function these virulence factors play in the respective pathogens. 

Most of the studies to identify the regions within the RTX toxins that provide species specificity or receptor specificity were performed when existing or engineered restriction digest sites were the tools used to generate chimeric proteins. Our knowledge would be greatly advanced with a three-dimensional RTX toxin structure, and more ideally, a structure of an RTX toxin together with a β_2_ integrin. With such critical advancements, mapping of functional domains could be refined with the use of in vitro gene synthesis to generate region/domain-specific mutants or even single amino acid substitutions across the proteins of interest to define the critical interacting domains. Screening peptides for inhibition of toxin binding or the cytolytic activity of the toxins based on the interacting domains could provide a route for therapeutic interventions against the relevant pathogens. Disruption of the secreted RTX toxin’s fatal interaction with host leukocytes may provide an opportunity for the primary host immune response to attack and clear the invading bacteria before a more severe and disseminated infection occurs.
